# Contract Development and Manufacturing Organizations (CDMO): are they needed in Brazil

**DOI:** 10.1186/1753-6561-8-S4-O3

**Published:** 2014-10-01

**Authors:** Dina Iezzi

**Affiliations:** 1 TherapureBiopharma Inc., Mississauga, Canada

## 

Life-altering biologic therapeutics are complex large molecules which include hormones, growth factors, interferons, vaccines, monoclonal antibodies, recombinant proteins and others. The time, cost and risk for advancing a biologic through the pipeline comes at a steep price. It is estimated that the development of a biologic therapeutic is as high as US$1.2 billion [1] versus the cost of developing a small molecule Innovator estimated at US$350 million [2]. Consequently, a common characteristic of most protein therapeutics is the relatively high cost.

Brazil's government is looking at measures to control the rising cost of healthcare with expenditures of $491 bn BRL ($220 bn USD)[3], along with $49.6 bn BRL ($21.6 bn USD)[4] for pharmaceutical drugs and $5.35 BRL ($2.4 USD)[5] for biologics. Despite the rising cost pressures, Alexandre Padilha, Minister of Health stated in the February 2012 Focus Report that "Brazil firmly believes in universal access and supports all initiatives that promote universal healthcare"[6].

The growing demand of biologics is an added financial stress on Brazil's National Public Healthcare System SUS (Sistema Único De Saude) which includes coverage of drug cost. With patent expiry approaching for several blockbuster biologics, Brazil is assessing multi-faceted programs and partnerships that facilitate the introduction of biosimilar therapeutics. The government initiative for the development of local biosimilars and improvement of national biologics capabilities, attempts to address cost savings measures to the healthcare system while reducing the risk of supply associated with imported branded biologics.

Through the coordination of the Ministry of Health and private and public companies, the Partnership for Productive Development (PDP) initiative was developed for the local manufacturing of 14 biological drugs seven of which are monoclonal antibodies (mAb)[7] that involve facilities with greater complexity and unique manufacturing processes.

The manufacturing process is important for all drugs, but especially for biological medicines, which are more complex structurally requiring a high degree of technology and scientific expertise to stabilize the complex molecular structures. These differences are important to take into account when understanding how the manufacturing process for a biological medicine can have an impact on product efficacy, quality and safety. Establishing "biosimilarity" to a reference product for a biosimilar drugs requires more preclinical and clinical testing than is usually necessary for generic small-molecule drugs because of the complexities of the molecular structure and manufacturing process of biologics.

Biosimilar development is a high-risk activity demanding significant resources with cost around US$75 - $250 million [8] versus the cost to develop a generic small molecule (US$1 million [9] - $3.5 million [10]). Additionally, building a biologics manufacturing facility is expected to be in the range of up to US$400 million [11]. One option to mitigate the high cost and risk of developing and manufacturing biosimilars is to partner with Contract Development and Manufacturing Organizations (CDMO). CDMOs specializing in the manufacturing of complex biologics can provide access to capacity with lower risk and investment and apply their scientific and technical expertise to serve as a strategic partner in the development and manufacturing of biosimilars.

References
